# Complex-Phase Steel Microstructure Segmentation Using UNet: Analysis across Different Magnifications and Steel Types

**DOI:** 10.3390/ma16237254

**Published:** 2023-11-21

**Authors:** Bishal Ranjan Swain, Dahee Cho, Joongcheul Park, Jae-Seung Roh, Jaepil Ko

**Affiliations:** 1 Department of Computer & AI Convergence Engineering, Kumoh National Institute of Technology, Gumi-si 39177, Republic of Korea; bishalswain@kumoh.ac.kr; 2 Research Institute of Science and Technology, Pohang-si 790660, Republic of Korea; daheecho@rist.re.kr (D.C.); ltpark@rist.re.kr (J.P.); 3 School of Materials Science and Engineering, Kumoh National Institute of Technology, Gumi-si 39177, Republic of Korea; jsroh@kumoh.ac.kr

**Keywords:** steel microstructure, phase fraction calculation, UNet segmentation, EBSD segmentation

## Abstract

The quantification of the phase fraction is critical in materials science, bridging the gap between material composition, processing techniques, microstructure, and resultant properties. Traditional methods involving manual annotation are precise but labor-intensive and prone to human inaccuracies. We propose an automated segmentation technique for high-tensile strength alloy steel, where the complexity of microstructures presents considerable challenges. Our method leverages the UNet architecture, originally developed for biomedical image segmentation, and optimizes its performance via careful hyper-parameter selection and data augmentation. We employ Electron Backscatter Diffraction (EBSD) imagery for complex-phase segmentation and utilize a combined loss function to capture both textural and structural characteristics of the microstructures. Additionally, this work is the first to examine the scalability of the model across varying magnifications and types of steel and achieves high accuracy in terms of dice scores demonstrating the adaptability and robustness of the model.

## 1. Introduction

Phase fraction calculation is a crucial step in materials science, serving as an indispensable tool for bridging the gap between a material’s composition, processing, microstructure, and properties [1]. This holds true especially for steel, whose properties are intricately interlinked with its complex microstructure encompassing phases like ferrite, bainite, pearlite, and martensite. Each phase distinctly impacts the material’s mechanical, thermal, and electrical attributes [2]. The accurate quantification of these phase fractions enables not only the prediction but also the manipulation of steel properties to meet specified performance requisites [3,4]. This becomes significantly important when steel is alloyed with various elements to augment its features. Therefore, phase fraction calculation is crucial in devising optimal alloy compositions, culminating in desired microstructures and properties [5]. The calculation of the distribution and volume fractions of these phases is crucial for forecasting key mechanical attributes such as strength, hardness, and toughness [6], making the precise annotation and segmentation of phase fraction calculation a practical imperative in steel material advancement.

**Figure 1:**
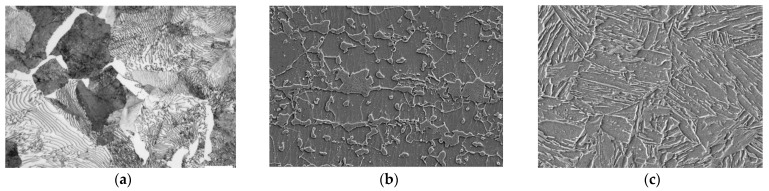
(**a**) OM image of normal tensile strength steel (520 Mpa), (**b**) SEM image of high-tensile strength steel (780 Mpa), and (**c**) SEM image of ultra-high-tensile strength steel (1180 Mpa).

Historically, phase annotation has been manually performed by experts who identify different phases in microstructural imagery, distinguishing them based on aspects like grain boundaries, color, contrast, and texture. While manual annotations provide accurate outcomes, they are labor-intensive and prone to human errors [7]. The emergence of machine learning and deep learning has positioned convolutional neural networks (CNNs) [8] as a promising substitute for this task, mainly focusing on relatively simpler microstructural images procured via optical microscopy (OM) or scanning electron microscopy (SEM) [9,10]. However, with an increase in the tensile strength of steels (the peak resistance displayed by a steel plate against pulling forces), its microstructure segmentation becomes increasingly complex, as shown in Figure 1.

As illustrated in Figure 1, an increase in tensile strength introduces greater ambiguity in both the phase structure and pattern, complicating the image annotation required for phase fraction calculation. Moreover, alloy steels exhibit further ambiguity in phase boundaries, complicating accurate phase identification. These challenges resemble those encountered in medical imaging where high-resolution high-precision segmentation models like UNet are typically used to find and segment the required elements. However, careful adaptation of such models is required to solve overfitting and to identify the intricate structures inherently present in steel microstructures.

The microstructures formed during the transformation-induced plasticity (TRIP-assisted) effect further complicate the segmentation task. The transformation entailing the deformation of retained austenite into martensite elevates work-hardening rates, enhancing the strength–ductility equilibrium [11]. The accurate identification of these microstructures for phase fraction calculation is crucial for evaluating the quality of Advanced High-Strength Steels (AHSS). Conventional methodologies like SEM analysis and Electron Backscatter Diffraction (EBSD) are commonly utilized for steel structure analysis, yet they are time-consuming, labor-intensive, and financially draining for extensive analysis [12,13], highlighting the necessity for alternative more efficient microstructure characterization methods. In this paper, we introduce a segmentation model for phase fraction calculation using a Deep Neural Network (DNN) on images of AHSS—a futuristic automotive steel material with tensile strengths surpassing 1 gigapascal (GPa).

**Figure 2:**
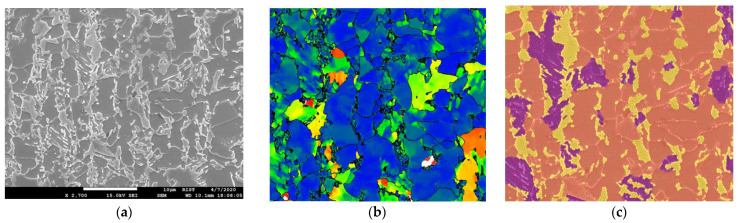
(**a**) SEM image of 2700× magnified alloy steel of 580 MPa, (**b**) the corresponding map generated from EBSD data, and (**c**) label image made using superpixel labeler along with EBSD map where purple corresponds to bainite, orange corresponds to ferrite, and yellow corresponds to martensite. The SEM image information present in the black bar was removed before training.

EBSD maps often show some inconsistencies that result in imprecise labeling and require manual correction. Manual supervision, while necessary, is time-consuming and prone to discrepancies due to variations in expert interpretations, fatigue, or subjective biases. To address these challenges, we have used our own superpixel [14] labeling software tool alongside EBSD maps to generate high-resolution and consistent label images, as illustrated in Figure 2. The superpixel labeling tool groups pixels into larger coherent superpixels based on similarity in color, texture, and other factors, thus providing a more efficient way to obtain accurately labeled images. Microscopic AHSS images present unique challenges in segmentation due to the intricate nature of steel microstructures and the similarities in appearance among different components under various magnifications [15]. In contrast to traditional image segmentation tasks where object boundaries are well-defined, the microstructures in alloy steel exhibit subtle distinctions and a variety of complex patterns. As can be seen in Figure 2c, the label image features three distinct labels corresponding to different phases of the steel microstructure: orange for ferrite, purple for bainite, and yellow for martensite. While ferrite and martensite phases exhibit definitive textures, bainite hardly presents any easily discernible pattern. The lack of definitive textural structures and edge delineation further complicates the task of phase identification.

Deep learning techniques, particularly CNNs, have shown promising results in various image segmentation tasks. The UNet architecture, a type of CNN, has been widely adopted for medical image segmentation due to its ability to capture both local and global contexts using an asymmetric encoder–decoder structure [16]. 

In steel microstructure segmentation, previous studies have explored various methodologies for achieving accurate classification and segmentation results. Traditional methods, such as the data mining techniques proposed by [17], have laid a solid foundation by extracting morphological features and employing SVMs for feature classification. While effective for simpler structures, these methods often struggle with the complex-phase steel microstructures due to the subtle differences between phases. Our approach extends these methods by leveraging a deep learning architecture that better captures the intricacies of complex-phase steel.

Pauly et al. [18] explored segmentation on contrasted and etched steel datasets acquired via SEM and LOM imaging, achieving modest accuracies. Our method builds upon this by implementing a UNet-based architecture that not only handles high-contrast variations but also adapts to the intricate textures found in high-strength alloys. Durmaz et al. [19] presented a multidisciplinary approach that combined specimen preparation with imaging, utilizing UNet to achieve an impressive 90% accuracy in lath–bainite segmentation. Inspired by their success, our work furthers the application of UNet in capturing the textural diversity of complex-phase steel microstructures, achieving similarly high accuracy rates. The microstructure of complex-phase steels typically consists of bainite, ferrite, and martensite or retained austenite [20] and the task of segmenting these phases is comparatively difficult due to the ambiguity between the structures of the phases. Azimi et al. [21] performed microstructure segmentation on SEM images containing bainite, ferrite, martensite, and pearlite using CNN and a max-voting scheme. Despite achieving high overall accuracy, their model struggled with certain phase segmentations, indicating a potential imbalance in the dataset. Our approach transcends this limitation by emphasizing the importance of not just texture but also the distinct shapes and structures within the microstructures. Via meticulous data augmentation and labeling, particularly using EBSD, our model demonstrates a nuanced understanding of these complex features.

Recent studies have begun leveraging UNet for the segmentation of dual-phase steel structures with clear class distinctions, simplifying the segmentation task [11,22]. In contrast, the complex-phase steels we investigate present a greater challenge due to the ambiguity in their microstructures. Our research addresses this by introducing advanced data augmentation techniques and precise EBSD-aided labeling that allows for more accurate phase distinction. The utilization of EBSD in annotation significantly refines the learning process, as it provides detailed pixel-wise structural orientation data, enhancing the segmentation accuracy beyond what conventional SEM or LOM data can achieve [19]. The work presented in [23] bears resemblance to our research objectives, laying down a foundational framework in the domain of steel microstructure segmentation. While their study also tries to tackle the segmentation of complex-phase steel, it relies on the MetalDAM [23] dataset, which lacks EBSD label images. Instead, they employ label images that were produced by using binary mask as pre-annotations before being modified by industry experts. This approach introduces subjectivity and potential inaccuracies into the annotation process. Additionally, their model does not appear to offer scalability across different magnifications or types of steel. Despite these limitations, their work has been a valuable source of inspiration for our own research. We further contribute by comparing the performance of our approach with the results from both the MetalDAM and UCHS [24] datasets.

The work of S. Bremuier et al. [25] also bears quite a resemblance to our research objectives of segmentation of complex-phase steels. Their research performs segmentation using band contrast (BC) and kernel average misorientation (KAM) as input into a deep learning model and achieves an accuracy of 92%. Contrastingly, our work shows that a similar result can be achieved without needing BC or KAM. Our model directly uses the input image generated from SEM and uses the labeled image generated using EBSD and a super-labeler as ground truth for the learning task. Moreover, [25] used low-carbon steels where our research delves into alloy steels. This distinction in steel type accounts for differences in the microstructure and properties. For instance, there seems to be a clear demarcation between structures or phases in low-carbon steels compared with alloy steels that might help in capturing the phases during the learning task. Distinct from earlier research, our proposed methodology focuses on phase fraction calculation using complex-phase steel microstructure segmentation by adapting pre-existing DNN models like UNet. We demonstrate that the deep learning model’s performance can be significantly improved by carefully selecting and tuning the parameters, rather than relying on pre-configurations. We also highlight the importance of data augmentation and precise labeling in segmentation tasks. To the best of our knowledge, we are the first to use only SEM-EBSD paired image maps as input for complex-phase segmentation and to conduct a scalability experiment, testing the performance of the model across different magnifications and steel-type images, thereby demonstrating its robustness and potential for practical application.

## 2. Materials and Methods

### 2.1. Motivation and Challenges

The phase fraction for a specific phase—be it ferrite, bainite, or martensite—is determined by the ratio of pixels representing that phase to the total pixel count in the image. This requires precise pixel-level annotation. While pixel-wise classification can be employed, it falls short of capturing the complex nuances of steel microstructures due to its lack of contextual awareness and semantic consistency. Therefore, segmentation emerges as a more fitting approach, offering the high precision and contextual understanding required for accurate phase fraction calculation.

The common practice of employing off-the-shelf models for segmentation tasks may not always yield optimal results due to the unique characteristics of specific problems. UNet models have gained popularity for their segmentation capabilities, characterized by a U-shaped design that balances context capture and precise localization [26,27,28,29]. While there are advanced variants of UNet, our research centers on customizing the foundational UNet model for the complex-phase steel microstructure segmentation task. The insights derived from this adaptation are not only crucial for our specific challenge but also serve as a foundational baseline that can be extended to other UNet variations, thereby enhancing the model’s broader applicability.

Data augmentation is vital for enhancing the performance of any DNN model, especially when using limited or imbalanced datasets. In steel microstructure segmentation, augmentation techniques must be carefully selected to capture the complex textures and structures of different phases [19,23]. There is also a need to address the class imbalance issue in our study. As can be seen in Figure 2, ferrite overshadows bainite and can lead to potential model bias if class imbalance is not properly addressed. Because of the labor-intensive nature of manual labeling and the substantial costs associated with generating EBSD images [30], evaluating the performance of the model across different magnifications and types of steel is crucial for assessing the generalizability of the model.

### 2.2. Materials and Usage

The steel used in this study is alloy steel commonly used in the automotive industry that exhibits exceptional strength, elongation, and formidability. Throughout the experiment, four different steel-type images and three types of magnifications were used.

- E-type: Alloy steel with a tensile strength of 580 MPa—Magnification: ×2700, ×3000, ×5000;
- A-type: Alloy steel with a tensile strength of 780 MPa—Magnification: ×5000;
- D3-type: Alloy steel with a tensile strength of 980 MPa—Magnification: ×5000;
- H2-type: Alloy steel with a tensile strength of 1108 MPa—Magnification: ×5000.

A total of 27 images were acquired using SEM and EBSD. Corresponding to each SEM image, we generated ground truth labels using EBSD, providing precise information about the distinct components and phases present within the steel images. Labeling of the SEM images was assisted with the use of a super-pixel labeler. Super-pixel segmentation groups pixels into larger coherent super-pixels based on similarity in color, texture, and other factors, thus providing a more efficient and accurate labeling process for our dataset. This approach, along with the precision of EBSD imaging and the detailed phase information from SEM, ensured the creation of a robust well-annotated dataset, serving as a solid foundation for training and evaluating our UNet segmentation model.

**Figure 3:**
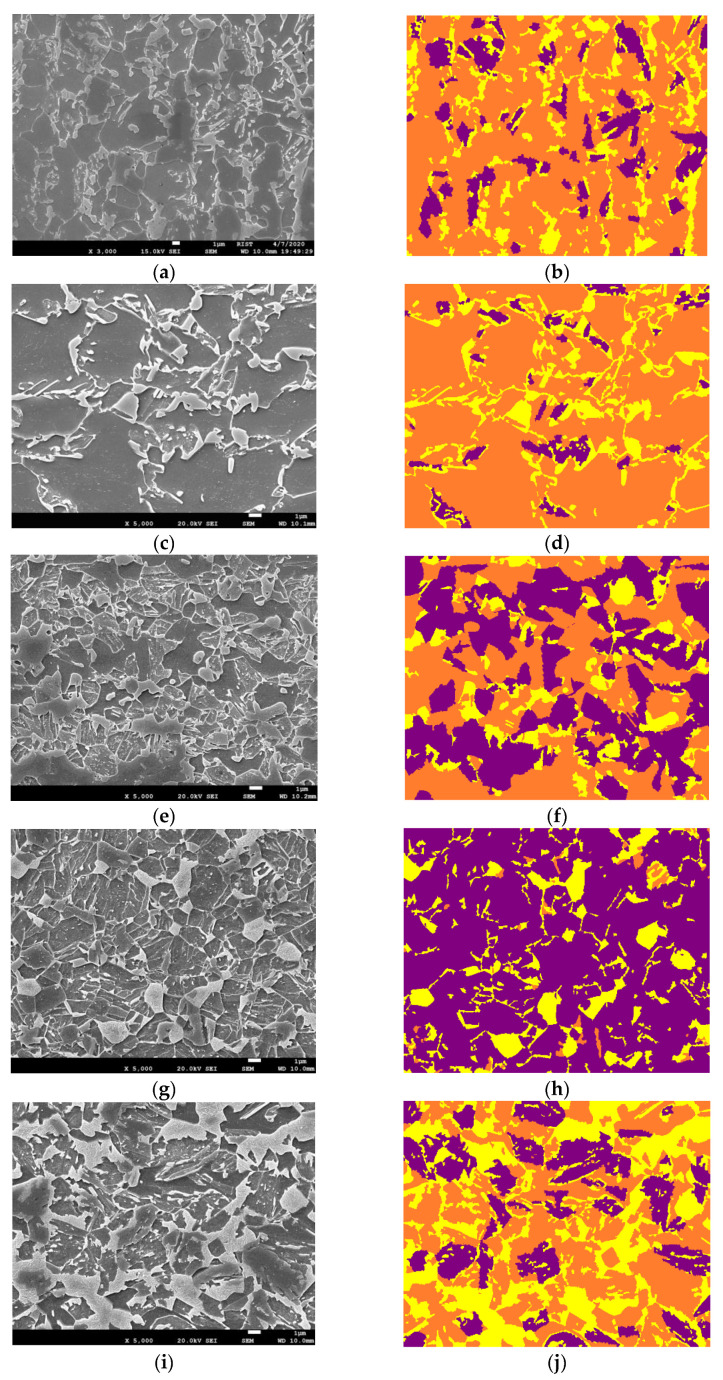
Images used for inferencing the model. (**a**,**c**) are E-type steel images at different magnifications than the images used during training, denoting scalability experiment 1. (**e**,**g**,**i**) have different magnifications and are different steel types than the images used during training, denoting scalability experiment 2. The label colors purple, orange, and yellow correspond to bainite, ferrite, and martensite, respectively. The SEM image information present in the black bar was removed before inferencing. (**a**) ×3000 E-type SEM, (**b**) ×3000 E-type Label, (**c**) ×5000 E-type SEM, (**d**) ×5000 E-type Label, (**e**) ×5000 A-type SEM, (**f**) ×5000 A-type Label, (**g**) ×5000 D3-type SEM, (**h**) ×5000 D3-type Label, (**i**) ×5000 H2-type SEM, (**j**) ×5000 H2-type Label.

In the training phase of our study, we used only ×2700 magnified E-type steel images to augment, train, and validate the model’s learning. We adopted the leave-one-out (LOO) cross-validation [31] test, where we isolated one image and trained the model using the rest. This ensures the robustness of findings of the model, allowing for an accurate performance assessment across diverse data subsets. To evaluate the model’s performance and its ability to generalize, we performed magnification scalability experiments using steel images of 3000× and 5000× magnifications. The ability to accurately segment images at these higher magnifications is crucial, as it enables the detailed analysis of complex microstructures that are typically observed at these magnified scales. Furthermore, to test the model’s versatility and its effectiveness across various steel types at different magnifications, we used A-, H2-, and D3-type alloy steel images at ×5000 magnification for different type and magnification scalability experiments. These images that were used during inferencing can be visualized in Figure 3. 

### 2.3. Methodology

**Figure 4:**
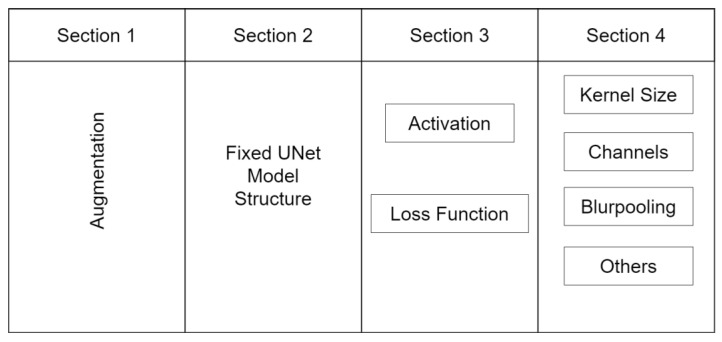
The four-step process of modifying the UNet-based model for steel image segmentation. The process includes performing problem-specific augmentations, working with fixed architecture components, optimizing loss and activation functions, and fine-tuning the model.

In the following section, we detail the methodology employed in this study. We conceptualize the usage of a deep learning model into four critical components, as depicted in Figure 4. These sections significantly influence the output of the model. The first section involves the application of appropriate data augmentations. The second section is the selection of a suitable model architecture. The third section involves the determination of the optimal loss function and activation functions. Lastly, the fourth section involves the fine-tuning of certain parameters to optimize the model or inclusion of special techniques to address specific issues.

#### 2.3.1. Data Augmentation and Rationale

Data augmentation techniques serve as essential tools for addressing the issue of data insufficiency by fabricating and expanding training sets via various transformations and adaptations of existing images. However, the choice of augmentations should align with the specific needs of the given problem. We employed several augmentation techniques which not only enhanced the diversity of our training dataset but also improved our model’s ability to generalize. Below are the augmentations that we used along with their proper rationale.

- Flip (x, y): We introduced horizontal (x-axis) and vertical (y-axis) flipping transformations to our images, creating mirror-like variations. This technique is beneficial as it accounts for varied orientations of steel microstructures, thereby ensuring our model learns to detect structures irrespective of their orientation;
- Rotation (0–90 degrees): Our augmentation suite includes random rotations of images within a 0 to 90-degree range. Given that steel structures in SEM images can exist in myriad orientations, this technique helps the model with rotation invariance, enabling better generalization;
- Zoom (1–2.5×): We applied random scaling to our images, ranging from 1 to 2.5 times their original size. This technique mimics the varied magnification levels in SEM images, thus preparing the model to handle different scales of steel structures;
- Intensity (0–10): We incorporated random intensity fluctuations into our images, introducing variations in brightness and darkness to diverse lighting conditions or material properties. This helps the model to better withstand intensity variations within steel images;
- Gamma (0–10): Adjusting the gamma value of our images served to enhance or suppress specific intensity levels. Gamma augmentation emulates contrast and brightness changes, thereby training the model to recognize features invariant to these differences;
- Contrast (HE): By applying histogram equalization (HE) to enhance image contrast, we redistributed pixel intensities to bolster overall contrast. This proved to be particularly beneficial in highlighting finer details and improving segmentation accuracy in low-contrast areas;
- Sliding Window: We incorporated the sliding window technique, dividing the larger input images into smaller patches of size 800 × 800 pixels with a stride of 15 pixels. Opting for larger patches facilitated the capture of complete structural elements, which would have been potentially obscured in smaller patches.

#### 2.3.2. Model Architecture

**Figure 5:**
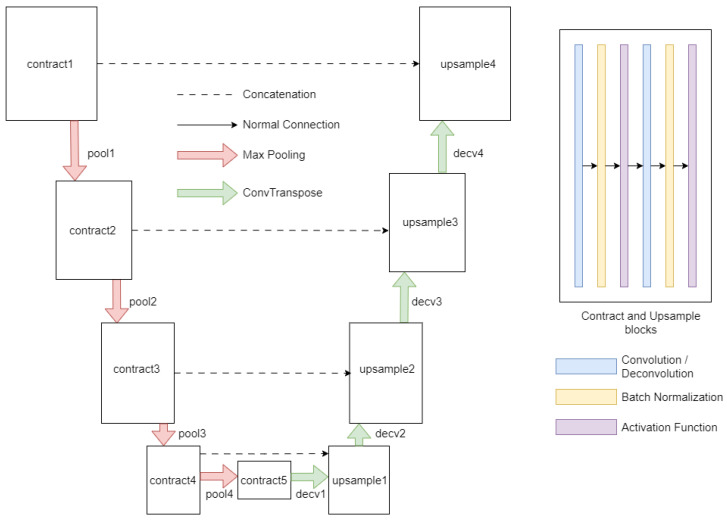
Scheme of the UNet architecture used in this work.

The second segment involves selecting an appropriate model for the segmentation task. Given that UNet-based models have consistently outperformed other model architectures, we recommend using a UNet-based model such as UNet++ [28], Unet3+ [32], R2Unet [33], etc. However, our aim is to adapt the UNet models specifically for the task of steel image segmentation. Therefore, we chose the barebone UNet model and demonstrated how it could be adapted for steel image segmentation. The UNet architecture, depicted in Figure 5, is designed as a combination of an encoder path and a decoder path.

The encoder path is responsible for extracting contextual information, while the decoder path contributes to precise localization. The encoder pathway is initiated with a contraction module (contract1) dedicated to applying a series of convolutional operations to discern low-level features from the input image. Subsequent modules, identified as pool1 through pool4, carry out pooling operations that systematically reduce the spatial dimensions of the feature maps, while simultaneously augmenting the number of channels. The subsequent contraction modules, identified as contract2 through contract5, perform additional convolutional operations to distill higher-level features.

Transitioning into the decoder pathway, the architecture aims to restore spatial information and refine the segmentation outcome. The first decoder module, designated as decv1, executes convolution-transpose or pixel-shuffling operations, hinging on the specified anti-aliasing type. This operation is followed by an up-sampling operation (upsample1) and a concatenation procedure, amalgamating features from the equivalent layer of the encoder path. This process is reiterated through the successive decoder modules, identified as decv2 through decv4, to further enhance the feature refinement. The final module in the decoder pathway (decv5) applies a convolutional operation to align the extracted features with the desired output classes. Various activation functions were also modularly integrated into the model architecture, such as ReLU [34], leaky ReLU [35], and Swish [36], and were used as per the needs of the experiment.

#### 2.3.3. Configuring Loss and Activation Functions

The appropriate configuration of loss and activation functions is crucial for the successful application of deep learning models to specialized problems. While these parameters often vary by domain, it is imperative to identify the most effective combinations for the problem at hand. In our study, we conducted extensive experiments to ascertain the optimal loss and activation functions tailored for the unique challenges of steel image segmentation.

##### Loss Function

Considering the unique challenges that we mentioned before in steel image segmentation, we used a combined loss function of Focal Loss [37], Jaccard Loss [38], and Multi-Scale Structural Similarity (MS-SSIM) Loss [39]. Each loss function addressed a specific issue resulting in the improved performance of the segmentation model.

- Focal Loss: This loss function is designed to address class imbalance by down-weighting inliers (easy examples) and focusing on outliers (hard examples). In our case, this is particularly useful as certain phases or structures like bainite are underrepresented in the dataset. By focusing on these challenging examples, the model is encouraged to learn features that distinguish these rare classes, thereby improving the overall segmentation performance;
- Jaccard Loss: Also known as Intersection over Union (IoU) loss, it measures the overlap between the predicted segmentation and the ground truth. This loss function is particularly useful for segmentation tasks as it directly optimizes for the quantity we are interested in the quality of the segmentation. In our case, Jaccard Loss helps the model to accurately capture the boundaries of different phases and structures, which is crucial for determining material properties;
- MS-SSIM Loss: The MS-SSIM loss function measures the structural similarity between the predicted and ground truth images. It is designed to capture the texture and structural information in an image. This is particularly useful in steel image segmentation where different phases and structures have distinct textures and shapes. By optimizing the MS-SSIM loss, the model learns to capture these textural and structural differences, thereby improving the segmentation of different phases and structures in the steel microstructures.

By combining these three loss functions, the model is encouraged to learn both structures and textures to segment the steel images accurately, considering the class imbalance, the need for accurate boundary delineation, and the importance of capturing textural and structural differences.

##### Activation Function

The activation function plays a pivotal role in introducing non-linearity into the learning process. This non-linearity enables the model to learn from the complex patterns and features in the images, which is essential for tasks like ours where the model needs to differentiate between subtle differences in textures and structures. We selected ReLU as the ideal activation function for our task. Although we also experimented using LeakyReLU and Swish, they did not perform well in comparison. ReLU introduces non-linearity without affecting the receptive fields of the convolution layer, which is beneficial to our task where preserving spatial information (like texture and structure) is crucial. On the other hand, Leaky ReLU and Swish, despite being improvements over ReLU in certain tasks, were not well-suited for our specific task of steel image segmentation.

#### 2.3.4. Optimizing Other Parameters and Experimental Techniques

The final part of the process involves fine-tuning the model. This includes making minor adjustments to the model architecture and hyper-parameters to improve performance. We employed the Adam optimizer [40], renowned for its adaptive learning rate efficiency. We selected a batch size of 16 and initial feature channel size of the UNet model as 16, which proved optimal for balancing memory utilization and training efficiency. We chose a batch size of 16 after considering a balance between computational efficiency and model performance. Larger batch sizes do indeed require more memory, which can be a limiting factor, especially when dealing with high-resolution images like those used in steel microstructure segmentation. A batch size of 16 allowed us to efficiently utilize our computational resources without compromising the training process’s stability. The choice of 16 initial feature channels in UNet was driven by the need to start with a sufficiently detailed feature representation while maintaining computational efficiency. It provides a balance between capturing the intricate details in the microstructures and ensuring that the model does not become overly complex and computationally demanding. It is a starting point that allows the network to progressively increase its complexity and learn finer details as it moves deeper into the architecture. The learning rate, set at 0.00001, ensured small step sizes for smoother convergence during training. This value was carefully chosen after a series of experiments and fine-tuning to achieve optimal performance.

- Input size: The input size of the image significantly impacts the performance of the model, especially in tasks like image segmentation where the goal is to capture intricate details and structures in the image. The larger input size allows the model to have more contextual information, improved learning of spatial hierarchies, and better handling of variability. Therefore, we used an input size of 800 × 800, and it performed better than 256 × 256 and 512 × 512 as expected. It is to be noted that increasing input size also increases the computational cost and memory requirements of the model;
- Kernel size: The kernel size in a convolutional layer determines the field of view that the model has when processing the input image and impacts the model’s ability to capture relevant features. A smaller kernel size, such as 3 × 3, means that the model is looking at a smaller portion of the image at a time. This can be beneficial for tasks where the important features are small and localized. For this reason, a larger kernel size, 7 × 7, was chosen which allowed the model to view a larger portion of the image at once;
- Blur pooling: To further optimize our model, we integrated BlurPool [41] into the architecture and tested three variations: up, down, and both up and down. Blur pooling aids in reducing the spatial resolution of feature maps while preserving critical information, potentially enabling the model to identify larger-scale patterns and mitigate overfitting [42];
- Adding boundary class: We attempted to add a boundary class to the labels by delineating edges between different phases. This approach aimed to enhance the ability of the model to accurately segment the boundaries of microstructures.

Despite the potential advantages, our experiments using blur pooling layers and the boundary class addition did not yield significant improvements in our model.

### 2.4. Experiment

#### Evaluation Metrics

In evaluating our deep learning model for steel microstructure segmentation, we have chosen metrics that not only reflect the model’s performance but also encapsulate the complexity and robustness required in the engineering discipline. Our selection is informed by the comprehensive analysis presented by Alavi et al. [43], which emphasizes the criticality of aligning performance fitness and error metrics (PFEMs) with the underlying physical mechanisms of the investigated system and ensuring robust validation practices. Four primary metrics were used for evaluation: Mean Pixel Accuracy, Dice Score, Class-wise Accuracy, and Phase Fraction.

1. Mean Pixel Accuracy (MPA): MPA offers a direct measure of the model’s accuracy at the pixel level across all classes. It serves as a foundational metric for preliminary assessment evaluating the pixel-level accuracy of predictions. The formula for calculating Mean Pixel Accuracy is given as:
  (1) $$Mean\;Pixel\;Accuracy=\frac{1}{n_{cl}}\underset{i}{\sum }\frac{n_{ij}}{t_{i}}$$
  where $n_{ij}$ is the number of pixels of class i predicted as class $j,\;n_{cl}$ is the total number of different classes, and $\;t_{i}$ is the sum of all pixels of class $i\;(t_{i}=\underset{j}{\sum }n_{ij}$).
2. Dice Score (F1 Score): The Dice Score, which measures the overlap between the predicted and ground truth segmentation masks, directly aligns with our task’s objective of high-quality segmentation due to its emphasis on boundary delineation. The Dice Score computes the overlap between the predicted segmentation mask (A) and the ground truth mask (B). The formula is:
  (2) $$Dice=\frac{2\times \left|A\cap B\;\right|}{\left|A\right|+\left|B\right|}$$
  where A is the predicted segmentation mask, B is the ground truth mask, |A| represents the number of pixels in A, and |B| represents the number of pixels in B. The numerator, 2 × |A ∩ B|, calculates the intersection between the predicted and ground truth masks multiplied by 2. The denominator, |A| + |B|, calculates the sum of the pixels in both masks. The Dice coefficient ranges from 0 to 1, with a value of 1 indicating a perfect overlap between the predicted and ground truth masks.
3. Class-wise Accuracy: Assessing the accuracy of individual classes allows us to address the model’s performance variations across different steel phases. This metric measures the accuracy of the model’s predictions for each individual class, providing a detailed understanding of its performance across different classes.
4. Phase Fraction: The Phase Fraction metric quantifies the proportion of each phase within a micrograph. This metric’s relevance is underscored by its direct correlation to material properties and to the objective of our task of phase fraction calculation. The phase fraction in a micrograph for a particular phase is the ratio of the number of pixels of that phase to the total number of pixels in the micrograph [5].

### 2.5. Experimental Setup

The setup uses a high-performance computing system equipped with an NVIDIA A6000 GPU and Intel i7 6700 CPU, running on the Ubuntu 22.10 operating system, and utilizing the PyTorch framework.

- Data Preparation: For the augmentation, training, and initial testing of the model, only ×2700 magnified E-type steel images were used. Inferences were performed on ×3000 and ×5000 E-type images for scalability experiment 1 and ×5000 A, D3, and H2 images were used for scalability experiment 2. The phases in label images were converted into class labels: purple (bainite) was denoted as 0, orange (ferrite) as 1, and yellow (martensite) as 2;
- Training and Validation Split: The augmented dataset was divided into training and validation sets using an 80/20 split ratio, where 80% of the augmented images were used for training and the remaining 20% for validation;
- Model Testing: After training was completed, testing was performed on the same magnification and same type of steel that was used while training (×2700 E-type steel). Two scalability experiments were performed for verifying the performance of the trained model on different magnifications and types of steel;
- Cross-Validation: LOO cross-validation was employed to ensure a robust assessment of the model’s performance. In this process, one image was sequestered from the training set at each fold, and the entire cycle of data preparation, splitting, training, and testing was reiterated.

## 3. Results

This section describes the results obtained using the experimental setup, categorized into three main groups: inferencing using the same steel type and magnification as the trained model, inferencing using the same steel type but different magnification levels (scalability 1), and inferencing using different steel types and different magnification levels compared to the trained model (scalability 2).

### 3.1. Comparison of Stock UNet and Augmentations

We present a tabular analysis of the performance of various UNet variants on the task of steel image segmentation. We investigate three distinct configurations: the Stock UNet Model with Random Augmentation (RA), the Stock UNet Model with our Custom Augmentation (CA), and UNet with modified parameters with Custom Augmentation. Random augmentation includes operations like random flips, rotations, scaling, and brightness adjustments during training.

Table 1 demonstrates the superiority of the Modified UNet with Custom Augmentation (Mod UNet + CA) across all steel types and magnifications, signifying the advantages of tailored model configurations and augmentation techniques. Notably, the Mod UNet + CA exhibits exceptional performance, with MPA values surpassing 84% and Dice Scores above 0.83 across all test scenarios. In stark contrast, the Stock UNet with Random Augmentation (RA) delivers suboptimal results, particularly at higher magnifications (×5000 E-type) where the complexity of the microstructures challenges the model’s recognition capabilities. This underscores the inadequacy of generic augmentations in capturing the nuanced features of complex-phase steel images. The Stock UNet with Custom Augmentation (CA) shows a moderate enhancement in performance over the RA variant. However, it still falls short compared to the Mod UNet + CA, highlighting the necessity of model customization to effectively handle domain-specific challenges.

### 3.2. Inference on Same Steel and Same Magnification

Upon isolating a test image from the training set, the trained model was subjected to an evaluation to measure its performance. As exhibited in Table 2, the model attained an overall pixel accuracy of 91.74%, coupled with a Dice Score of 0.9158. This high level of accuracy indicates an impressive overlap between the predicted segmentation and the actual microstructures.

**Figure 6:**
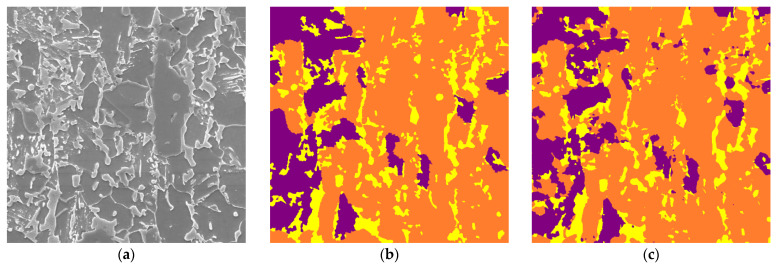
(**a**) ×2700 magnified E-type SEM test image. (**b**) Corresponding label image using EBSD. (**c**) Image predicted using our model. The label colors purple, orange, and yellow correspond to bainite, ferrite, and martensite, respectively.

A class-wise accuracy breakdown provides further insight into the model’s performance across different phases. The model demonstrates robustness in identifying ferrite with an accuracy of 96.1%, indicating its proficiency in segmenting this prevalent phase. Martensite and bainite, while posing more of a challenge due to their intricate structures, still achieved accuracies of 83.7% and 81.3%, respectively. These figures are especially significant considering the complexity of these microstructures and the difficulties inherent in their segmentation. The test SEM image, corresponding label image and the image predicted by our model is shown in Figure 6.

The results, particularly the high Dice Score, affirm the model’s capability to discern between the intricate textures and boundaries of the steel microstructures accurately. This is crucial for practical applications where material properties are inferred from microstructural composition.

### 3.3. Inference on Same Steel with Different Magnification—Scalability 1

**Figure 7:**
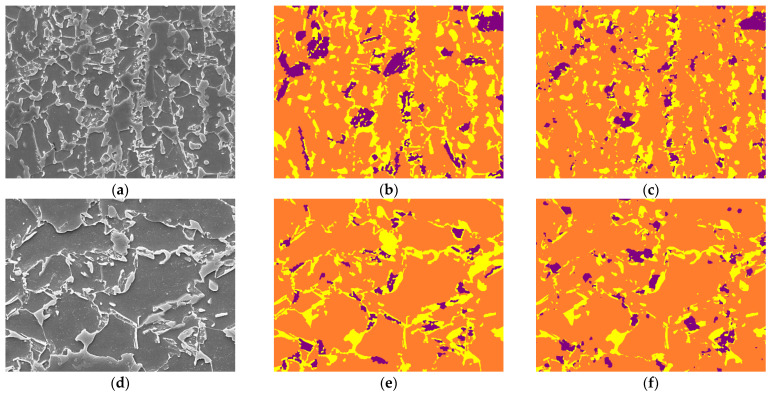
(**a**–**c**), respectively, represent ×3000 magnified E-type SEM test image, corresponding label, and image predicted using our model; (**d**–**f**), respectively, represent ×5000 magnified E-type SEM test image, corresponding label, and image predicted using our model. The label colors purple, orange, and yellow correspond to bainite, ferrite, and martensite, respectively.

Images of the same steel type (E-type) but at different magnification levels were used for inference. As depicted in Table 3 and illustrated in Figure 7, the model achieved Pixel Accuracies of 84.11% and 89.29% for ×3000 and ×5000 magnifications, respectively. The enhanced accuracy at ×5000 magnification underscores the model’s adeptness in handling higher-resolution images, which often present more detailed microstructural information.

The Dice Scores of 0.8389 and 0.8892 further validate the model’s efficacy in segmenting the microstructures accurately at different magnifications. Notably, the Accuracy Per Class and Phase Fraction metrics provide a deeper perspective on the model’s performance across various classes and phases. The Error Margin in Phase Fraction reveals a marginal discrepancy between the predicted and actual phase compositions, suggesting areas where the model’s precision can be further improved.

### 3.4. Inference on Different Steel and Different Magnification—Scalability 2

**Figure 8:**
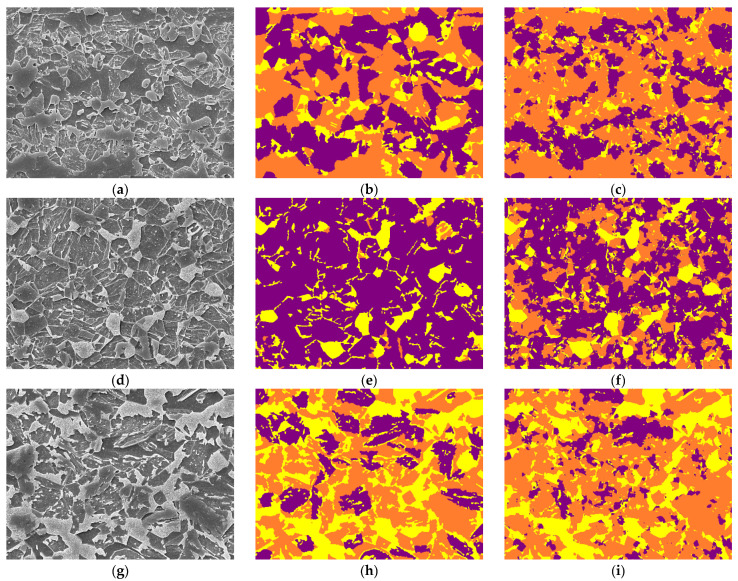
(**a**–**c**) represent ×5000 magnified A-type SEM test image, corresponding label, and predicted image, respectively; (**d**–**f**) represent ×5000 magnified D3-type SEM test image, corresponding label, and predicted image, respectively; (**g**–**i**) represent ×5000 magnified H2-type SEM test image, corresponding label, and image predicted using our model, respectively. The label colors purple, orange, and yellow correspond to bainite, ferrite, and martensite, respectively.

Scalability experiment 2 involved inferencing on A-type, D3-type, and H2-type steel images at ×5000 magnification. Table 4 and Figure 8 detail these results. The model exhibited competent performance across these different steel types and magnifications, with accuracies of 77.78%, 68.32%, and 73.92% for A-type, D3-type, and H2-type, respectively. The Dice Scores further corroborate the model’s ability to generalize well to steel types not encountered during training.

In particular, the variation in Accuracy Per Class and Phase Fraction across different steel types is noteworthy. These metrics illuminate the model’s adaptability to different microstructural compositions and complexities. The Error Margin in Phase Fraction, though present, remains within acceptable limits, reaffirming the model’s robustness in diverse scenarios.

### 3.5. Comparison on Other Datasets

To further validate the effectiveness of our proposed method and benchmark its results, we compared its performance with other established models, namely PixelNet [44], UNet, and UNet++, on two different datasets: UHCS and MetalDAM. The results of this comparison are presented in Table 5.

Table 5 presents the MPA performance comparison of different segmentation models, including PixelNet, UNet, UNet++, and our proposed method, on two datasets: UHCS and MetalDAM. The results are evaluated based on the accuracy metric. Our proposed method outperforms the other models in both datasets, achieving an accuracy of 95.79% on UHCS and 94.21% on MetalDAM.

## 4. Discussion

The experimental results from this study provide valuable insights into the calculation of the phase fraction using image segmentation of steel micrographic images. This section provides an analysis and interpretation of these results, addressing the strengths and limitations of our approach, investigating the factors influencing performance variations, discussing the encountered challenges, and outlining potential future work and improvement areas.

### 4.1. Experimental Result Analysis and Interpretation

In this study, we evaluated different aspects of a segmentation model, performed experiments on complex-phase EBSD images, performed experiments using various configurations while keeping an overall fixed UNet structure including activation functions, blur pooling, loss functions, and incorporation of a boundary class [detailed in Appendix A], and conducted scalability experimentation using different magnifications and steel types. Several factors contributed to the observed performance of the model.

- The augmentation of data was a significant aspect of the model performance, and it should be carefully performed keeping the objective in sight;
- The combined loss function focused on outlier examples and encouraged the model to learn and distinguish rare classes. It not only identified different structures but also different textures and shapes of different phases;
- As can be observed from Table A2 present in Appendix A, the ReLU activation function performed better than other counterparts. The ReLU activation function outputs a range from 0 to positive infinity, effectively nullifying negative values during forward propagation. This introduces sparsity into the network, thereby aiding in the mitigation of overfitting
- BlurPool layers, also known as anti-aliasing layers, are typically used to reduce high-frequency artifacts in the feature maps. The benefits of BlurPool layers are often most pronounced in tasks where high-frequency noise is a significant issue, such as in certain types of image classification tasks. However, in the case of microstructure steel image segmentation, the primary challenge is not necessarily high-frequency noise, but rather the complex and subtle differences between different phases of the steel. Therefore, the addition of BlurPool was effective at the same scale and in the same type of steel but was ineffective at different scales and in different types, as shown in Table A3 of Appendix A;
- The introduction of a boundary class is often used in segmentation tasks to help the model distinguish the transition areas between different classes, especially when the boundaries are ambiguous. But our experimentation results (Table A4 in Appendix A) showed that the inclusion of a boundary class did not produce expected outcomes. The boundaries between different phases might be highly complex and not easily captured by a simple boundary class. The transitions might involve subtle changes in texture and structure that require more sophisticated modeling approaches.

### 4.2. Challenges, Limitations, and Scope for Future Works

There are various challenges that can be faced when performing microstructure segmentation in the current study and that can lead to future research directions.

- Limited Data Availability: A major challenge encountered in this study is the limited amount of training data. The training dataset might not sufficiently capture the diversity and complexity of real-world steel images. This limitation is particularly relevant when considering the variability in imaging conditions, such as differences in contrast and working distances in SEM images. Incorporating a wider range of imaging conditions into the dataset could help improve the model’s robustness across different scenarios.
- Impact of Surface Treatment: Surface treatments, such as polishing with various grits of sandpaper, can significantly alter the microstructure’s appearance in microscopic images. This study did not explicitly account for the effects of different surface treatments on prediction accuracy. Future work could explore how these treatments impact the model’s ability to accurately segment and calculate phase fractions, potentially addressing a crucial aspect of practical applicability.
- Presence of Microstructural Defects: The functionality of the prediction model in the presence of microstructural defects, such as small cracks, is not thoroughly explored in this study. Cracks and other defects can influence the segmentation accuracy by altering the appearance of phases in the steel microstructures.
- Complexity of Phase Differentiation: Differentiating between various phases in AHSS images is inherently challenging. There are instances where a microstructure may appear similar yet have different phase compositions. Capturing not only the textural information but also the shape and structure of the phase microstructures becomes a complex task for the model. The absence of a clear demarcation between different phases of steel in EBSD images adds another layer of complexity to the segmentation task.

## 5. Conclusions

This study has presented a comprehensive approach to the segmentation of microstructures in steel images for the calculation of phase fraction which is critical for understanding material properties. We have demonstrated the potential of adapting the UNet architecture, a model originally designed for medical image segmentation, to the specific challenges of steel image segmentation. Our approach has shown that the performance of the model can be significantly improved by carefully selecting and tuning the model parameters, rather than using off-the-shelf configurations. We followed a systematic approach like grid search for finding the optimal values of hyper-parameters, where we tested a range of values for each parameter and observed their impact on model performance. We have also highlighted the importance of data augmentation in addressing the challenges posed by the intricate and complex nature of steel microstructures. By applying appropriate augmentations, we have been able to enhance the model’s ability to capture both the texture and structure of different phases in the microstructures.

Our work has further underscored the importance of precise labeling in segmentation tasks. We have used complex-phase EBSD images for segmentation, a method that, to our knowledge, has not been used before in this context of segmentation of alloy steels. Our work also underscores the importance of configuring the parameters of UNet models like activation function, loss function, input size, kernel size, and other techniques that can be used to adapt the model to the problem statement. Our use of a combined loss function tailored to the need of the problem addressed the issues of capturing complex microstructures in images.

Finally, we are the first to conduct such scalability experiments, testing the model at different magnifications and on different steel-type images. The results have shown that our approach is more robust as the model performs relatively well even on the unseen data, demonstrating that the model has learned the structures and textural information well.

## Figures and Tables

**Table 1:** Performance comparison using MPA (dice) of basic and modified UNet models with our augmentation strategies.

Variations	×3000 E-Type	×5000 E-Type	×5000 A-Type	×5000 D3-Type	×5000 H2-Type
Stock UNet + RA	58.85 (0.5743)	28.82 (0.2768)	46.29 (0.4601)	39.9 (0.3842)	44.48 (0.4292)
Stock UNet + CA	70.01 (0.6876)	78.75 (0.7803)	64.29 (0.6393)	55.61 (0.5328)	63.44 (0.6233)
**Mod UNet + CA**	**84.11 (0.8389)**	**89.29 (0.8892)**	**77.78 (0.7731)**	**68.32 (0.6809)**	**73.92 (0.7376)**

Mod UNet+CA outperforms when using Stock UNet for steel image segmentation.

**Table 2:** MPA (dice) along with accuracy per phase in the test image.

Image Type	Test Image Accuracy
Pixel Accuracy	91.74 (0.9158)
Martensite	83.7
Ferrite	96.1
Bainite	81.3

**Table 3:** Different metrics of predicted images of ×3000 and ×5000 magnification of E-type steel.

Metrics	×3000 E-Type	×5000 E-Type
Pixel Accuracy	84.11	89.29
Dice Score	0.8389	0.8892
Accuracy Per Class	(68.11, 93.77, 45.33) *	(70.87, 96.57, 41.73) *
Phase Fraction [label]	(0.1982, 0.7029, 0.0989) *	(0.176, 0.7789, 0.0451) *
Phase Fraction [Predicted]	(0.1514, 0.7904, 0.0583) *	(0.1282, 0.8196, 0.0522) *
Error Margin of Phase Fraction	(−0.0438, +0.0875, −0.0406) *	(−0.0478, +0.0407, +0.0071) *

* represent values of three elements—martensite, ferrite, and bainite, respectively.

**Table 4:** Different metrics of predicted images of ×5000 magnification and A, D3, H2-type of steels.

Metrics	A-Type	D3-Type	H2-Type
Pixel Accuracy	77.78	68.32	73.92
Dice Score	0.7731	0.6909	0.7376
Accuracy Per Class	(51.04, 92.11, 65.45) *	(79.64, 24.41, 66.89) *	(78.52, 88.11, 37.62) *
Phase Fraction [label]	(0.1441, 0.4281, 0.4278) *	(0.1742, 0.0184, 0.8074) *	(0.3404, 0.4313, 0.2283) *
Phase Fraction [Predicted]	(0.1, 0.5683, 0.3317) *	(0.1796, 0.236, 0.5844) *	(0.2819, 0.573, 0.1451) *
EM of Phase Fraction	(−0.0441, 0.1402, −0.0961) *	(0.0054, 0.2176, −0.223) *	(−0.0585, 0.1417, −0.0832) *

* represent values of three elements—martensite, ferrite, and bainite, respectively.

**Table 5:** Performance comparison in MPA of PixelNet, UNet, UNet++, and our proposed method on UHCS and MetalDAM datasets.

Dataset	PixelNet	UNet	UNet++	Our Proposal
UHCS	90.77	91.15	91.15	95.79
MetalDAM	84.07	86.33	87.04	94.21

Our proposal outperforms previous related studies in terms of MPA.

## Data Availability

The data presented in this study are available on request from the corresponding author. The data are not publicly available due to privacy restrictions.

## Appendix A

Contains a list of experiments performed either to find better parameters for the UNet model or the effects of certain techniques that were implemented to achieve optimal performance.

1. Loss Functions

Model results with different loss functions—Focal Loss, Jaccard Loss, and Combined Loss are detailed in Table A1.

**Table A1:** Pixel accuracy and dice measurements for models with different loss functions.

Loss	×3000 E-Type	×5000 E-Type	A-Type	D3-Type	H2-Type
Focal	81.97 (0.7972)	87.01 (0.8676)	74.68 (0.7116)	61.75 (0.5997)	70.89 (0.6764)
Jaccard	83.35 (0.8168)	86.06 (0.8459)	75.94 (0.7231)	65.34 (0.6392)	72.47 (0.7076)
**Combined Loss**	**84.11 (0.8389)**	**89.29 (0.8892)**	**77.78 (0.7731)**	**68.32 (0.6809)**	**73.92 (0.7376)**

The results are represented in the form of MPA (dice). Combined Loss performs comparatively better than Focal and Jaccard loss.

The results, as summarized in the table, were measured in terms of pixel accuracy (dice score) for various types of steel. Among the tested loss functions, Combined Loss demonstrated superior performance across all types of steel, outperforming the use of only Focal or Jaccard Loss. This finding emphasizes the importance of selecting an appropriate loss function tailored to the specific characteristics of the task, as it can significantly influence the model’s ability to accurately segment steel microstructures.

2. Activation Functions

Different activation functions were examined to determine their impact on the model performance. ReLU performed better, as illustrated in Table A2.

**Table A2:** Pixel accuracy and dice measurements for models with different activation functions.

Activation	×3000 E-Type	×5000 E-Type	A-Type	D3-Type	H2-Type
**ReLU**	**84.11 (0.8389)**	**89.29 (0.8892)**	**77.78 (0.7731)**	**68.32 (0.6809)**	**73.92 (0.7376)**
Leaky ReLU	84.77 (0.8452)	88.53 (0.8831)	75.19 (0.7493)	59.59 (0.5855)	69.69 (0.6938)
Swish	84.21 (0.8396)	88.67 (0.8842)	72.41 (0.7217)	56.54 (0.5649)	70.00 (0.6973)

The results are represented in the form of MPA (dice). The bold text shows that ReLU performs better than other activation functions.

We evaluated the impact of different activation functions on the model’s performance across various types of steel. The activation functions examined included ReLU, Leaky ReLU, and Swish. Comparatively, ReLU demonstrated superior performance across all types of steel, with notable achievements in pixel accuracy and dice measurements.

3. Effects of Blur Pooling

We explored the effects of blur pooling layers on the performance of our model, particularly focusing on various steel types and scales. Our experiments tested different combinations of blur pooling, including Down, Up, and Down + Up, alongside a baseline model without any blur pooling (None). The results are presented in Table A3, offering insights into the impact of these layers on model performance.

**Table A3:** Pixel accuracy and dice measurements for models with different blur pooling layers.

Blur Pool	×3000 E-Type	×5000 E-Type	A-Type	D3-Type	H2-Type
**None**	84.11 (0.8389)	**89.29 (0.8892)**	**77.78 (0.7731)**	**68.32 (0.6809)**	**73.92 (0.7376)**
Down	83.53 (0.8329)	87.69 (0.8743)	70.83 (0.7061)	52.5 (0.5227)	68.02 (0.6777)
Up	**84.41 (0.8416)**	87.93 (0.8768)	76.73 (0.7648)	45.04 (0.4479)	69.0 (0.6875)
Down + Up	84.3 (0.8405)	87.51 (0.8726)	74.5 (0.7425)	40.24 (0.3999)	66.78 (0.6653)

The results are represented in the form of MPA (dice). Bold shows the highest accuracy achieved among different blur pool techniques across different magnifications and types of steel images.

We observed that the inclusion of blur pooling layers did not consistently improve the model’s performance across all steel types and scales. Notably, for the ×3000 E-type images, the models with blur pooling, particularly the ‘Up’ configuration, showed comparable performance to the baseline. This suggests that, for certain image scales, blur pooling may have a stabilizing effect on the model, potentially reducing high-frequency noise which can be more pronounced in higher-resolution images.

However, for other types of steel, especially those at different scales (e.g., ×5000 E-type, A-type, D3-type, H2-type), the blur pooling layers did not contribute to performance enhancement. In fact, the baseline model without blur pooling consistently outperformed models with these layers. This discrepancy in effectiveness could be attributed to the inherent characteristics of the different steel types and their microstructures. It is possible that the microstructural details in these steel types are either less affected by high-frequency noise or that the blurring effect may be obfuscating critical microstructural details necessary for accurate segmentation.

4. Inclusion of a Boundary Class

The introduction of a boundary class in the classification task was intended to improve boundary localization, enhancing discrimination, reducing ambiguity, and enabling fine-grained analysis.

**Table A4:** Accuracy and dice measurements for models with boundary class and non-boundary class.

Class	×3000 E-Type	×5000 E-Type	A-Type	D3-Type	H2-Type
w/o Boundary Class	84.11 (0.8389)	89.29 (0.8892)	77.78 (0.7731)	68.32 (0.6809)	73.92 (0.7376)
w/ Boundary Class	80.04 (0.7979)	84.94 (0.8469)	70.95 (0.707)	62.53 (0.6228)	65.02 (0.6477)

The results are represented in the form of MPA (dice).

The results, presented in the table, compare the performance of models with and without the boundary class across various types of steel. Surprisingly, the models without the boundary class outperformed those with the boundary class in terms of accuracy and dice measurements. This outcome suggests that the introduction of a boundary class did not contribute to the improvement of the segmentation task in this context.

5. Learning Rate

Experiments were conducted on a wide range of learning to find out the best possible hyper-parameter. To identify the optimal learning rate, we conducted experiments across a range from 0.01 to 0.000001. The performance of our model at different learning rates is captured in Table A5, which details the Mean Pixel Accuracy (MPA) and Dice scores across various steel types.

When keeping a high learning rate (0.01), the model exhibited suboptimal performance, particularly on the A-type, D3-type, and H2-type steels. The lower performance in these cases suggests that the model might have been stuck in local minima, missing the global optimal solution. As the learning rate decreased, we observed a gradual improvement in performance. The model achieved its best performance at a learning rate of 0.00001, as evidenced by the highest MPA and Dice scores across all steel types. Further lowering of the learning rate to 0.000001 resulted in a slight decrease in performance, indicating the onset of diminished returns and potentially slower convergence.

**Table A5:** Accuracy and dice measurements for various learning rates.

Learning Rates	×3000 E-Type	×5000 E-Type	A-Type	D3-Type	H2-Type
0.01	82.78 (0.8249)	87.03 (0.8679)	51.60 (0.5138)	26.48 (0.2584)	51.69 (0.5092)
0.001	72.42 (0.7081)	76.02 (0.7491)	59.47 (0.5901)	47.92 (0.4627)	59.20 (0.5869)
0.0001	78.94 (0.7743)	84.26 (0.8378)	67.15 (0.6582)	54.63 (0.5298)	68.65 (0.6759)
**0.00001**	**84.11 (0.8389)**	**89.29 (0.8892)**	**77.78 (0.7731)**	**68.32 (0.6809)**	**73.92 (0.7376)**
0.000001	81.08 (0.8024)	86.92 (0.8589)	75.63 (0.7487)	65.23 (0.6396)	67.06 (0.6592)

The results are represented in the form of MPA (dice). The bolded text denotes the optimal learning rate that produced comparatively better results.

6. Cross-Validation Results

Leave-one-out (LOO) cross-validation was carried out to check the performance of our model across various subsets of the dataset. LOO cross-validation is an ideal technique for use with datasets with a limited number of samples, as it allows for a comprehensive assessment of the model’s performance on nearly the entire dataset. Based on the subset of images that were chosen, the performance of the model varied. The results of the LOO cross-validation are presented in Table A6. The table shows the Mean Pixel Accuracy (MPA) and Dice scores for each cross-validation fold across different steel types and magnifications.

**Table A6:** Accuracy and dice measurements for different magnifications and types of steel across cross-validation subsets.

Cross-Validation	×3000 E-Type	×5000 E-Type	A-Type	D3-Type	H2-Type
CV-1	83.93 (83.37)	89.10 (88.70)	79.93 (79.38)	64.02 (63.57)	77.43 (76.86)
CV-2	85.59 (85.23)	87.02 (86.41)	77.30 (76.75)	63.35 (62.87)	75.64 (75.25)
CV-3	84.47 (83.79)	91.56 (91.08)	77.69 (77.14)	71.47 (70.89)	72.26 (71.91)
CV-4	83.89 (83.38)	92.28 (91.75)	81.27 (80.59)	70.93 (70.60)	75.45 (75.02)
CV-5	82.68 (82.21)	86.48 (86.17)	72.72 (72.15)	71.84 (71.28)	68.83 (68.38)
**Average**	**84.11 (0.8389)**	**89.29 (0.8892)**	**77.78 (0.7731)**	**68.32 (0.6809)**	**73.92 (0.7376)**

The results are represented in the form of MPA (dice). The bold text denotes the average of the cross-validation results.
